# Supplementary material for: Effect of Plant Growth Regulators on Cotton Seedling Root Growth Parameters and Enzyme Activity
Source: Plants (Basel). 2022 Nov 3;11(21):2964. doi: 10.3390/plants11212964 (PMC9657821; doi:10.3390/plants11212964)
Supplement: Supplementary file 1 [file plants-11-02964-s001.zip › plants-1956278-supplementary.pdf]

Table S1. Eigenvalues and contributions of principal components.

	PC1	PC2	PC3
Eigenvalues	3.118	1.974	1.070
percent variance, %	44.549	28.193	15.292
Cumulative contribution rate, %	44.549	72.742	88.034
root length	0.932	-0.221	-0.142
root surface area	0.976	-0.004	0.055
root volume	0.792	0.150	0.303
root mean diameter	-0.758	0.400	0.124
soluble protein	-0.255	-0.935	0.042
POD	0.171	0.930	-0.157
SOD	-0.031	0.061	0.956
Principal Component Name	morphogen factor	protein content factor	enzyme activity factor

Table S2. Rank of the scores of principal components.

Treatment	morphogen factor	sort	protein content factor	sort	enzyme activity factor	sort	overall ratings	sort
Z619-G1	1.13	10	0.07	14	-1.82	27	-0.31	16
Z619-G2	-1.3	21	-0.42	20	-1.44	24	-1.6	26
Z619-G3	-1.02	18	-3.2	27	-1.49	26	-2.89	27
Z619-S1	2.38	2	-0.25	19	0.18	11	1.17	5
Z619-S2	2.23	3	-1.54	24	1.5	2	1.1	6
Z619-S3	1.59	7	2.48	1	0.73	7	2.43	1
Z619-P1	1.61	6	1.12	6	0.64	9	1.71	3
Z619-P2	-0.26	15	0.63	9	0.11	12	0.24	12
Z619-P3	-0.33	16	2.36	2	-1.1	23	0.47	11
Z27-G1	1.73	5	-0.15	16	-1.46	25	0.06	14

Z27-G2	1.46	8	0.23	12	-0.15	18	0.78	10
Z27-G3	0.23	13	-2.34	26	-0.06	16	-1.1	23
Z27-S1	0.19	14	0.71	8	0.69	8	0.8	9
Z27-S2	1.92	4	-0.04	15	0.76	6	1.34	4
Z27-S3	-1.81	23	-1.44	23	0.26	10	-1.51	25
Z27-P1	-1.69	22	-1.35	21	2.62	1	-0.21	15
Z27-P2	-2.92	26	0.5	10	1.08	3	-0.68	19
Z27-P3	-2.17	25	0.22	13	-0.05	15	-1.01	21
Z39-G1	1.35	9	2.13	3	0.1	13	1.82	2
Z39-G2	-1.05	19	-0.18	17	-0.87	20	-1.06	22
Z39-G3	1.02	11	-1.88	25	1.05	4	0.1	13
Z39-S1	2.82	1	-0.23	18	-0.99	22	0.81	8
Z39-S2	-0.67	17	-1.35	22	0.04	14	-1	20
Z39-S3	-1.15	20	0.93	7	-0.93	21	-0.58	18
Z39-P1	0.57	12	0.3	11	0.94	5	0.91	7
Z39-P2	-2.05	24	1.53	4	-0.24	19	-0.38	17
Z39-P3	-3.84	27	1.15	5	-0.11	17	-1.42	24
